# Exploitation of gamma delta T cells in cancer immunotherapy as combined antigen-presenters and cancer cell killers

**DOI:** 10.1186/2051-1426-2-S3-P18

**Published:** 2014-11-06

**Authors:** Gitte Holmen Olofsson, Manja Idorn, Wajid Khan, Mads Hald Andersen, Bernhard Moser, Per thor Straten

**Affiliations:** 1 Center for Cancer Immune Therapy, Herlev, Denmark; 2 Cardiff Institute of Infection & Immunity, Cardiff CF14 4XN, United Kingdom

## 

The human Vγ9Vδ2 T cells are a unique T cell type, and recent studies of the biology of Vγ9Vδ2 T cells emphasize the potential exploitation of these cells in immunotherapy of cancer. Vγ9Vδ2 T cells exhibit dual functionality in that they are both antigen-presenting cells and cytotoxic towards cancer cells. We have been able to show that Vγ9Vδ2 T cells can kill cancer cells from various cancer types such as breast cancer, leukemia cancer lines and malignant melanoma, with a significantly increased killing upon treatment of the cancer cells with Zoledronic acid. In addition, cross presentation of antigens was also confirmed by using flow cytometry and chromium release assays. Furthermore, Vγ9Vδ2 T cells were also able to induce a conventional CMV-specific αβ-T cells response/culture. Unique to these findings is that it is the same γδ T cells that exhibit both functionality as APC and cancer killers. This combined with the ease of expanding Vγ9Vδ2 T cells in vitro to billions of cells, makes Vγ9Vδ2 T cells an attractive alternative to conventional antigen-presenting cells, such as dendritic cells. Moreover, a cell that kills tumor targets and concurrently induces a response against the tumor cell it kills, holds great potential for clinical use. We are currently setting up in vivo experiments using the NOG mouse model to study the in vivo capacity of Vγ9Vδ2 T cells to delay tumor growth.

